# An In Vitro Study for Evaluating Permeability and Metabolism of Kurarinone

**DOI:** 10.1155/2020/5267684

**Published:** 2020-09-13

**Authors:** Youfa Qin, Yongkun Zhu, Xiaoyan Xue, Guanghui Zhou, Huibo Li, Jian Wang

**Affiliations:** ^1^Department of Clinical Pharmacy, SSL Central Hospital of Dongguan City, Dongguan, China; ^2^Rehabilitation Department of Traditional Chinese Medicine, Dongguan Third People's Hospital, Dongguan, China; ^3^Department of Pharmacy, Peking University Third Hospital, Beijing, China; ^4^School of Pharmaceutical Engineering, Shenyang Pharmaceutical University, Shenyang, China

## Abstract

Kurarinone is a major component found in the dried roots of *Sophora flavescens* Ait. that participates in vital pharmacological activities. Recombinant CYP450 supersomes and liver microsomes were used to study the metabolic profiles of kurarinone and its inhibitory actions against cytochrome P450 (CYP) and UDP-glucuronosyltransferase (UGT) enzymes. 100 *μ*M of kurarinone strongly inhibited more than 90% of UGT1A1, UGT1A6, CYP1A2, and CYP2C9. CYP1A2 and CYP2D6 played important roles in catalyzing the biotransformation of kurarinone. Moreover, metabolism of kurarinone considerably differs among species, and metabolic characteristics were similar between monkey and human. Kurarinone demonstrated moderate permeability at values of pH 4.0 and 7.4. Our findings offer a clearer idea to understand the pharmacological and toxicological mechanisms of kurarinone.

## 1. Introduction

**Figure 1:**
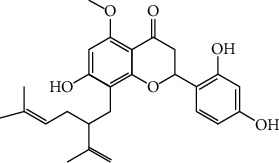
Structure of kurarinone.

The dry root of *Sophora flavescens* Ait. has been an important species in traditional Chinese medicine since the Qin and Han dynasties. *S*. *flavescens* has been documented in editions of the Pharmacopoeia of the People's Republic of China since 1977. Novel dosage forms in terms of tablets, capsules, and injections were developed, recently [1]. Currently, *S*. *flavescens* is widely used to treat dysentery [1], fever [2], skin and mucosal ulcers [3], diarrhea [4], and reduce edema by inducing diuresis [5]. Besides, *S*. *flavescens* is known to exhibit varied pharmacological properties including antitumor [6, 7], anti-inflammatory [8], and antibacterial [9, 10] activities. Kurarinone (showed in Figure 1) is a major constituent of *S*. *flavescens* with the content ranging from 4.79 mg/g to 16.07 mg/g [11]. Kurarinone exhibits antitumor [12, 13], anti-inflammatory [8], antioveractive bladder [14], antioxidant [15], and antityrosinase [16] activities. It also induced remarkable cytotoxicity in primary rat hepatocytes and HL-7702 cells [17, 18].

Cytochrome P450 (CYP) and UDP-glucuronosyltransferase (UGT) are important phase-I and -? drug-metabolizing enzymes that actively participate in the metabolism of more than 90% of currently available drugs [19]. Inhibition or induction of CYPs or UGTs might cause potential drug-drug interactions (DDIs). *S*. *flavescens* induces CYP3A expression by activating the pregnane X receptor (PXR) [20]. *S*. *flavescens* extract has been found to reduce blood theophylline concentration in rats by inducing hepatic CYPs including CYP1A2, CYP2B, CYP2E1, and CYP3A [21]. Several other studies reported that *S*. *flavescens* extract dose-dependently inhibited human hepatic CYP2C8, CYP2C9, CYP1A2, CYP2C19, CYP2B6, and CYP3A4 [2, 22, 23]. Such varying experimental outcomes may be due to the complexity of *S*. *flavescens* extracts. Although kurarinone is a marker compound of *S*. *flavescens*, the interactions between former and CYPs or UGTs are yet to be investigated earlier.

Currently, the parallel artificial membrane permeation assay (PAMPA) is used as a rapid in vitro assay of passive biomembrane permeation in the drug discovery stage [24]. This technique was developed by Kansy et al. [25] and had been originally established to rapidly predict passive permeability through the gastrointestinal tract [26]. Nevertheless, the permeability of kurarinone has not yet been reported.

In this study, the liver microsomes and recombinant human supersomes were used to explore the interaction of kurarinone and UGTs or CYPs, which would contribute to its safe clinical usage and minimize the occurrence of DDIs.

## 2. Materials and Methods

### 2.1. Chemicals and Reagents

Kurarinone (>98%) was purchased from BioBioPha Co., Ltd. (Kunming, China). Glucose-6-phosphate, glucose-6-phosphate dehydrogenase, NADP^+^, D-glucose-6-phosphate, Tris-HCl, 7-hydroxycoumarin, 4-methylumbelliferone (4-MU), 4-methylumbelliferone-*β*-D-glucuronide, uridine 5′-diphosphoglucuronic acid (UDPGA; trisodium salt), phenacetin, diclofenac, dextromethorphan, chlorzoxazone, testosterone, (*S*)-mephenytoin, sulfaphenazole, quinidine, clomethiazole, furafylline, ketoconazole, and omeprazole were purchased from Sigma Aldrich (St. Louis, MO, USA). Metabolites of the probe substrates including 6-hydroxylated chlorzoxazone (2E1), O-deethylated phenacetin (CYP1A2), 4′-hydroxylated diclofenac (2C9), 4′-hydroxylated (*S*)-mephenytoin (2C19), O-demethylated dextromethorphan (2D6), and 6*β*-hydroxylated testosterone (3A4) were provided from the Research Institute for Liver Disease Co., Ltd. (Shanghai, China). Pooled liver microsomes from humans (HLMs), monkeys (MLMs), rabbits (RAMs), rats (RLMs), mouse (MOMs), dogs (DLMs), and minipigs (PLMs) were provided by the Research Institute for Liver Disease Co., Ltd. (Shanghai, China). Recombinant human supersomes (UGT1A1, UGT1A3, UGT1A6, UGT1A7, UGT1A8, UGT1A9, UGT1A10, UGT2B4, UGT2B7, UGT2B15, CYP1A2, CYP2C9, CYP2D6, CYP2E1, CYP3A4, and CYP2C19) were obtained from BD Gentest Corp. (Woburn, MA, USA).

### 2.2. Analytical Instruments and Conditions

The HPLC analysis was performed using the Shimadzu LC-20AB system (Shimadzu, Kyoto, Japan) equipped with the Hypersil BDS C18 column (Dalian Elite Analytical Instruments Co., Dalian, China; 4.6 × 150 mm, 5 *μ*m). The mobile phase consisted of (A) water containing 0.1% formic acid and (B) acetonitrile with a flow rate of 1.0 mL/min. A gradient elution program was performed: 0.0–11.0 min, 40%–60% B; 11.0-12.00 min, 60%–90% B; 12.0–15.0 min, 90% B; 15.0–16.0 min, 90%–40% B; and 16.0–22.0 min, 40% B. Kurarinone and its metabolites were detected at 280 nm.

### 2.3. In Vitro Incubation Systems in Liver Microsomes or Recombinant CYP Supersomes

Samples were prepared in a total volume of 200 *μ*L, containing 0.3 mg/mL protein in human liver microsomes or 15 nM protein in recombinant human supersomes, probe substrates or 50 *μ*M kurarinone, 10 mM MgCl_2_, 10 mM glucose-6-phosphate, and 1 unit/mL of glucose-6-phosphate dehydrogenase. The reactions were activated by adding NADPH following preincubation at 37°C for 5 min. The reactions were maintained at 37°C for 1 h and terminated by adding 200 *μ*L ice-cold acetonitrile. The supernatant (10 *μ*L) collected from the samples that were centrifuged (20,000 g, 20 min, 4°C) was injected into the HPLC system for analysis. Control incubations devoid of NADPH or kurarinone were performed parallelly to ensure that the produced metabolites were NADPH- or kurarinone-dependent.

### 2.4. Inhibitory Effect of CYP Activities by Kurarinone

RLMs were used to assess the inhibitory effects of kurarinone (100 *μ*M) on six different human isoforms (CYP1A2, CYP2C9, CYP2D6, CYP2E1, CYP3A4, and CYP2C19). The probe substrates used in this experiment were 40 *μ*M phenacetin for CYP1A2, 10 *μ*M diclofenac for CYP2C9, 25 *μ*M dextromethorphan for CYP2D6, 120 *μ*M chlorzoxazone for CYP2E1, 35 *μ*M testosterone for CYP3A4, and 20 *μ*M (*S*)-mephenytoin for CYP2C9 as described earlier [27]. The reaction system has been described in Section 2.3. Kinetic parameters including IC_50_ and *K*_*i*_ were determined and analyzed for CYP isoforms whose activities were strongly inhibited by more than 90%. Dixon and Lineweaver–Burk plots were formed to confirm the reversible inhibition type, and a second plot of slopes from the Lineweaver–Burk plot over the concentrations of kurarinone was used to estimate the *K*_*i*_ value. All incubations were performed in triplicates, and mean values were used for analysis.

### 2.5. Inhibition of 4-MU Glucuronidation Assay

4-MU, a nonspecific probe substrate for UGT isoforms, was used to explore the inhibition of UGT isoforms by kurarinone. The mixture (200 *μ*L total volume) contained recombinant UGTs (final concentrations: 0.125, 0.05, 0.025, 0.05, 0.025, 0.05, 0.05, 0.25, 0.05, and 0.2 mg/mL for UGT1A1, UGT1A3, UGT1A6, UGT1A7, UGT1A8, UGT1A9, UGT1A10, UGT2B4, UGT2B7, and UGT2B15, respectively), 5 mM UDPGA, 5 mM MgCl2, 50 mM Tris-HCl buffer (pH 7.4), and 4-MU in the absence or presence of various concentrations of kurarinone. The concentrations of 4-MU were as follows: 110 *μ*M for UGT1A1 and UGT1A6, 1200 *μ*M for UGT1A3 and UGT2B4, 15 *μ*M for UGT1A7, 750 *μ*M for UGT1A8, 30 *μ*M for UGT1A9, 80 *μ*M for UGT1A10, 350 *μ*M for UGT2B7, 250 *μ*M for UGT2B15, and 2000 *μ*M for UGT2B17. Kurarinone was dissolved in methanol, and the final concentration of methanol was 0.5% (*v*/*v*). Reactions were initiated by adding UDPGA, and incubations were carried out at 37°C in a shaking waterbath for 60 min. The reactions were terminated by addition of 200 *μ*L of ice-cold acetonitrile containing 7-hydroxycoumarin (100 *μ*M) as the internal standard. The incubation mixture was then centrifuged at 20,000 ×*g* for 20 min to obtain the supernatant, and an aliquot of supernatant (20 *μ*L) was transferred to an autoinjector vial for HPLC analysis.

### 2.6. Reaction Phenotyping Assays

Recombinant human supersomes (CYP1A1, CYP1A2, CYP1B1, CYP2A6, CYP2B6, CYP2C8, CYP2C9, CYP2C19, CYP2D6, CYP2E1, CYP3A4, CYP3A5, and CYP4F2) were used to valuate the CYP enzyme participating in kurarinone metabolite formation. The incubation for each CYP isoform was carried out as described in Section 2.3. Kurarinone (20 *μ*M) was incubated with each of the recombinant CYPs (15 nM) at 37°C for 30 min, and plausible metabolites were monitored.

### 2.7. Chemical Inhibition Study

Chemical inhibition studies were carried out by adding various human CYP inhibitors to the incubation mixture of kurarinone (20 *μ*M) prior to the addition of the NADPH-generating system. HLMs were utilized as metabolic enzymes. The period of incubation and conditions are detailed in Section 2.3.

### 2.8. Kinetics Study

Various concentrations of kurarinone (1–200 *μ*M) were incubated with RLMs, MLMs, PLMs, RAMs, CYP1A2, and CYP2D6 to estimate kinetic parameters. The rest of the other conditions remained the same as described in Section 2.3. Preliminary experiments evidenced that the formations of metabolites occurred in a linear range of reaction time and concentrations of microsomes. The apparent *K*_*m*_ and *V*_max_ values were calculated from nonlinear regression analysis of experimental data according to the Michaelis–Menten equation, and the results were represented graphically by Eadie–Hofstee plots. All incubations were carried out as triplicate independent experiments, and kinetic constants were recorded as mean ± SD.

### 2.9. Prediction of In Vivo Hepatic Clearance

The following equations were utilized to predict the in vivo hepatic clearance of kurarinone in humans and rats [28].

(1) $$\begin{array}{c} {\text{CL}}_{\text{int in vitro}}=\frac{V_{\max}}{K_{m}}, \end{array}$$

(2) $$\begin{array}{c} {\text{CL}}_{\text{int in vitro}}={\text{CL}}_{\text{int in vitro}}\cdot \text{SF,} \end{array}$$

(3) $$\begin{array}{c} {\text{CL}}_{H}=\frac{Q_{H}\cdot f_{u}\cdot {\text{CL}}_{\text{int in}}}{Q_{H}+f_{u}\cdot {\text{CL}}_{\text{int in vivo}}}, \end{array}$$

where the SF (scaling factor) represents the milligrams of microsomal protein per gram of the liver multiplied by the grams of liver weight; CL_int_ is the intrinsic metabolic clearance; CL_*H*_ is hepatic clearance; *f*_*u*_ is the free fraction in the blood (no data were available for kurarinone; thus, *f*_*u*_ was arbitrarily proposed to be 1); and *Q*_*H*_ is the hepatic blood flow. The CL_*H*_ of kurarinone was calculated using equations (1)–(3). The physiological parameters for calculating the intrinsic clearance in rats and humans are described as follows: the amounts of microsomal protein were 44.8 mg and 48.8 mg of protein/g of the liver; the liver weight per kilogram of body weight values were 40 g and 25.7 g; and the liver blood flow values were 55.2 mL/min/kg and 20.7 mL/min/kg, respectively [29].

### 2.10. Molecular Docking

Molecular docking analysis was performed to further assess the molecular mechanism of interaction between kurarinone and CYPs. The X-ray structures of CYP 1A2 (PDB code 2HI4), 2C9 (PDB code 3QM4), and 2D6 (PDB code 4WNU) were obtained from RCSB Protein Databank (http://rcsb.org/). Molecular docking evaluations were performed with AutoDock 4.02. Schrodinger Maestro software was utilized for graphic display. The protein structures were prepared through ProteinPrep wizard within Schrodinger package, and the energy minimization was completed through the external Tripos forcefield. The cluster analysis with AutoDock results was performed to determine best poses of kurarinone within investigated CYP sites.

### 2.11. PAMPA Permeability Study

The PAMPA method was used to measure the membrane permeability values, as described by Singh et al. in an earlier study [30]. Drug samples were dissolved in DMSO and then diluted to 10 mM concentrations as a stock solution. 300 *μ*L diluted drugs solution was filled in the PAMPA plate as the “donor” wells, and the 96-well filter plate with a synthetic phospholipid membrane was then placed on the donor wells. The “acceptor” wells were filled with 200 *μ*l of buffer solution, and the PAMPA instruments were incubated at room temperature for 5 h. Kurarinone permeability was assessed in quadruplicate at pH 4.0 and 7.4. Naproxen was taken as a high permeability marker and furosemide as a low permeability marker. At the end of the incubation, kurarinone and the marker compounds in the donor and acceptor wells were determined by HPLC, and permeability was calculated.

## 3. Results

### 3.1. Metabolic Profiling of Kurarinone in Different Liver Microsomes

**Figure 2:**
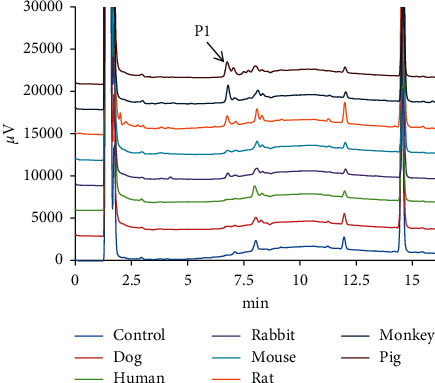
Chromatogram of kurarinone and its metabolites incubated with HLMs, DLMs, MLMs, RAMs, PLMs, and RLMs, respectively. Kurarinone (20 *μ*M) was incubated with liver microsomes (0.5 mg/mL) at 37°C for 60 min.

After kurarinone (20 *μ*M) was incubated for one hour with different liver microsomes such as HLMs, RLMs, RAMs, PLMs, MOMs, MLMs, and DLMs, HPLC was utilized to confirm the biotransformation of kurarinone. As showed in Figure 2, a new peak (*P*_1_) was detected at 6.77 min compared with that of the control group, which was inferred as the main metabolite of kurarinone owing to its high peak area for all species. The formation of metabolite was time-, NADPH-, and microsome-dependent.

### 3.2. Inhibitory Effects of Kurarinone against UGT and CYP Activities

**Figure 3:**
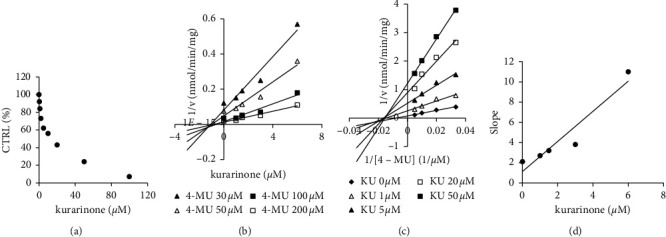
Inhibition kinetic analysis of kurarinone (KU) toward recombinant UGT1A1-catalyzed 4-MU glucuronidation. (a) Concentration-dependent inhibition of UGT1A1 by kurarinone. (b) Dixon plot of kurarinone inhibition of UGT1A1-catalyzed 4-MU glucuronidation. (c) Lineweaver–Burk plot of kurarinone inhibition of UGT1A1-catalyzed 4-MU glucuronidation. (d) Secondary plot of slopes from the Lineweaver–Burk plot versus kurarinone concentrations to determine *K*_*i*_. Every data point represents the mean of triplicate determinations.

**Figure 4:**
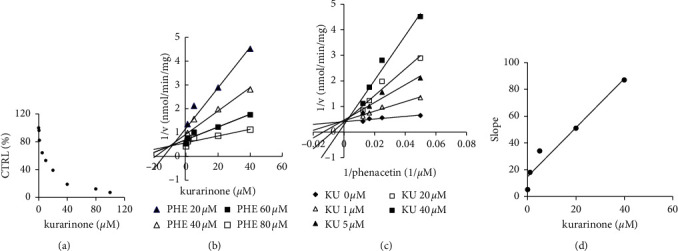
(a) Inhibitory effects of kurarinone (KU) on phenacetin (PHE) o-deethylation activity (CYP1A2). (b) Dixon plot of the inhibition effect of kurarinone on phenacetin o-deethylation (CYP1A2). (c) Lineweaver–Burk plot of the inhibitory effect of kurarinone on phenacetin o-deethylation (CYP1A2). (d) Secondary plot of slopes from the Lineweaver–Burk plot versus kurarinone concentrations. Each data points represent mean of two replicates.

100 *μ*M kurarinone inhibited the activities of UGT1A1, UGT1A6, CYP1A2, and CYP2C9 by more than 90%. Inhibition kinetic parameters of UGT1A1 and CYP1A2 were established. Kurarinone displayed concentration-dependent inhibitions against UGT1A1 and CYP1A2 with IC50 values of 13.64 *μ*M and 10.02 *μ*M, respectively (Figures 3(a) and 4(a)). Dixon (Figures 3(b), and 4(b)) and Lineweaver–Burk plots (Figures 3(c), and 4(c)) demonstrated that UGT1A1 and CYP1A2 inhibition by kurarinone was fitted well by noncompetitive and competitive inhibition, respectively. The *K*_*i*_ values were calculated to be 13.04 *μ*M and 8.13 *μ*M for UGT1A1 and CYP1A2 by a second plot of the slopes from the Lineweaver–Burk plots versus kurarinone concentrations (Figures 3(d), and 4(d)). Kurarinone exhibited competitive inhibition against CYP1A2 and noncompetitive inhibition against UGT1A1.

### 3.3. CYP Isoforms Involved in Catalyzing the Formation of Metabolite

**Figure 5:**
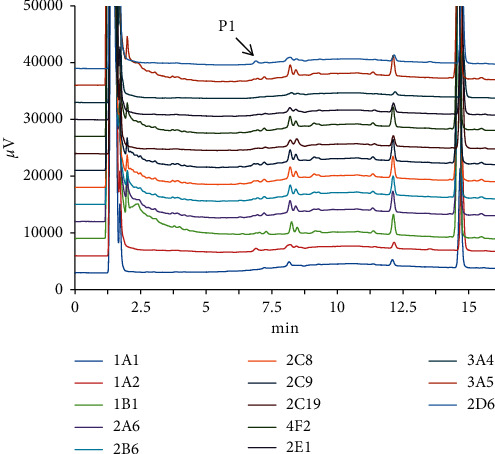
Chromatogram of kurarinone and its metabolites incubated with recombinant CYP450 supersomes. Kurarinone (20 *μ*M) was incubated with recombinant CYP450 supersomes (15 nM) at 37°C for 30 min.

After kurarinone was incubated with different liver microsomes, its main metabolite (*P*_1_) was determined. Kurarinone was incubated with 13 cDNA-expressed human CYP isoforms for confirming the CYP isoform involved in metabolizing kurarinone.  Figure 5 indicates that CYP1A2 and CYP2D6 played a significant role in catalyzing the formation of *P*_1_, whereas the other enzymes hardly contributed in metabolizing kurarinone.

**Figure 6:**
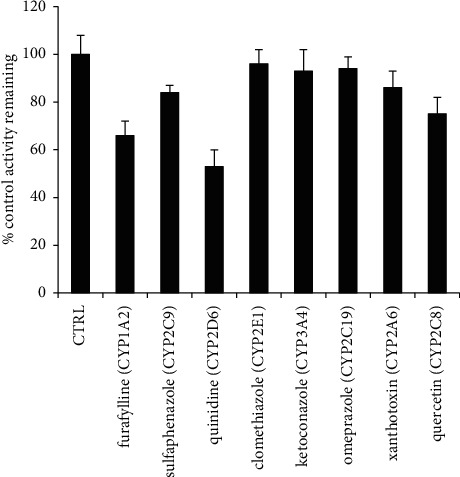
Inhibition assays of kurarinone metabolism by selective CYP450 inhibitors in MLMs. Results were mean ± SD of at least 3 separate assays.

The results were confirmed through the chemical inhibition study. Kurarinone was incubated with selective chemical inhibitors of the eight CYP isoforms in HLMs, and the results showed that furafylline (CYP1A2 inhibitor) and quinidine (CYP2D6 inhibitor) remarkably inhibited the formation of *P*_1_ compared with other CYP isoform inhibitors (Figure 6).

### 3.4. Enzyme Kinetics Study

**Figure 7:**
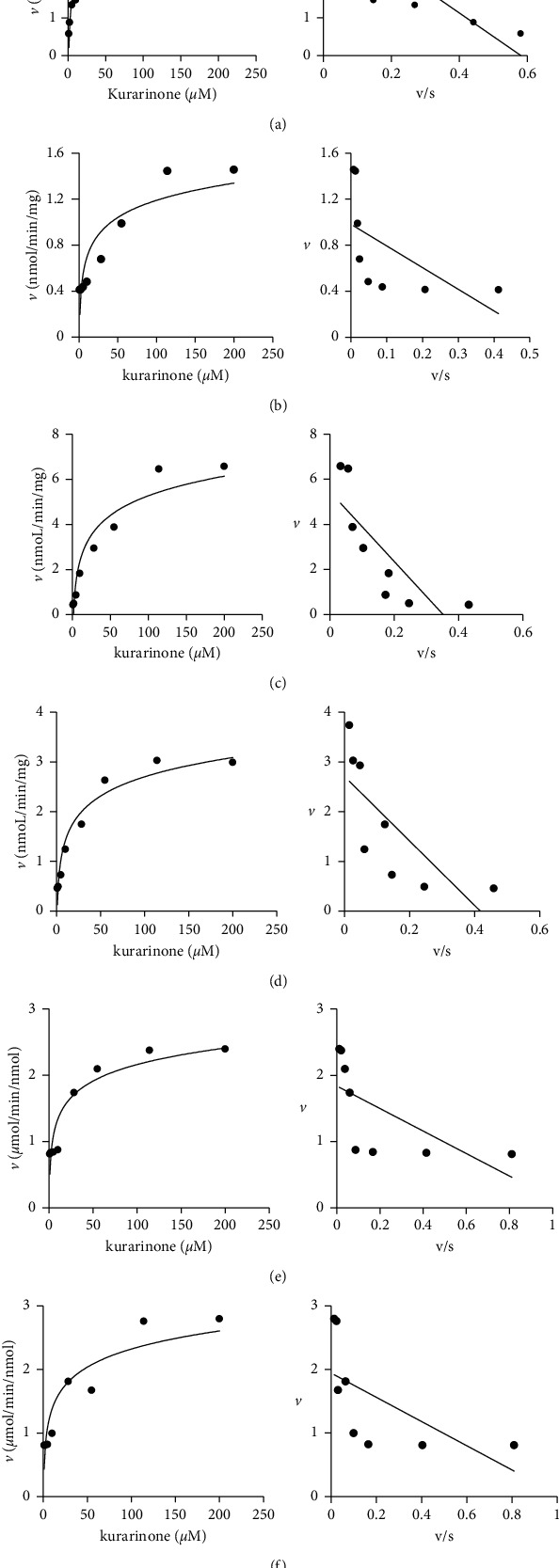
Michaelis–Menten plots and Eadie–Hofstee plots of kurarinone (1–200 *μ*M) in (a) MLMs, (b) HLMs, (c) PLMs, (d) RLMs, (e) CYP1A2, and (f) CYP2D6. Each data point represents the mean of triplicate determinations.

As shown in Figure 7, the metabolic profiles of kurarinone (1–200 *μ*M) in RLMs, MLMs, PLMs, HLMs, CYP1A2, and CYP2D6 exhibited typical monophasic Michaelis–Menten kinetics, which were further confirmed by Eadie–Hofstee plots. The kinetic parameters (*K*_*m*_, *V*_max_, and CL_int_) were calculated using *P*_1_ data and are summarized in Table 1.

### 3.5. Prediction of In Vivo Hepatic Clearance in Human and Rat

CL_*H*_ was calculated using the kinetic parameters of *P*_1_ generated from nonlinear regression in HLMs and RLMs, and the results were 16.82 mL/min/kg and 46.01 mL/min/kg body weight for humans and rats. The percentages of CL_*H*_ versus hepatic blood flow (*Q*_*H*_) values for human and rat were 81.27% and 83.35%, respectively.

### 3.6. Molecular Modeling

**Figure 8:**
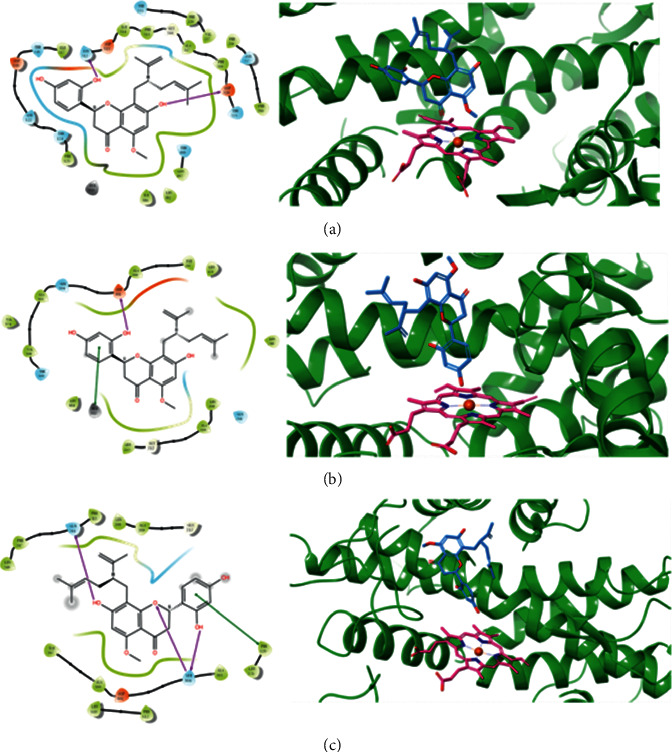
Docking simulations of kurarinone with human CYP 1A2 (a), CYP2C9 (b), and CYP2D6 (c). H-bonds are shown as arrows. CYP protein is displayed as green ribbons, HEME as pink sticks, and kurarinone as blue sticks.

CYP2D6 and CYP1A2 were observed to be two important metabolic enzymes catalyzing the biotransformation of kurarinone, while kurarinone strongly inhibited the activities of CYP2C9 and CYP1A2. Molecular docking evaluations were performed to further verify the interaction between kurarinone and CYP2D6, CYP2C9, and CYP1A2 at a molecular level. Kurarinone was found to bound within the binding pockets of CYP1A2, CYP2C9, and CYP2D6, forming H-bonds with Asn312 and Asp320 of CYP1A2 (Figure 8(a)), Asp301 of CYP2C9 (Figure 8(b)), and Gln244 and Ser304 of CYP2D6 (Figure 8(c)), separately. Hydrophobic residues surrounding kurarinone were also observed within the investigated CYPs, including Ile117, Phe125, Phe319, Leu382, Ile386, and Leu497 within the CYP1A2 pocket, Leu208, Leu213, Leu248, Ile297, and Leu484 within the CYP2C9 pocket and Phe120, Leu121, Leu208, Phe243, Phe247, Phe248, and Ile297 within the CYP2D6 pocket, respectively.

### 3.7. PAMPA Permeability of Kurarinone

Drugs are primarily known to be absorbed in the small intestines, wherein the pH may vary from acidic to neutral and slightly basic. In this study, the PAMPA assay was carried out at pH 4.0 and 7.4, respectively. The PAMPA permeability results of kurarinone and permeability markers are showed in Table 2. Kurarinone exhibited moderate permeability at pH 4.0 and 7.4.

## 4. Discussion

Owing to its various pharmacological activities, kurarinone will be coadministered with other drugs. Hence, assessment of potential DDIs is an essential process in the clinical development of kurarinone. Both Food and Drug Administration (FDA) and European Medicines Agency (EMA) affirmed that *C*_max_/*K*_*i*_ ratio was calculated as an indicator of in vivo DDI potential. The interaction likely occurred if the *C*_max_/*K*_*i*_ value of the inhibitor is greater than 1, possibly occurred if the ratio is between 0.1 and 1, and unlikely occurred if below 0.1 [31]. Kurarinone potently inhibited CYP1A2 with the Ki value of 8.13 *μ*M in RLMs. The *C*_max_ value of kurarinone in rats was recorded to be 1668.01 ng/mL [32], and *C*_max_/*K*_*i*_ value was calculated to be 0.46, indicating that the in vivo adverse effects possibly occur due to the *C*_max_/*K*_*i*_ value of kurarinone against CYP1A2. Thus, potential metabolism-based DDIs might occur during coadministration of kurarinone with other CYP1A2 substrate such as tizanidine [33]. Nevertheless, it is noteworthy that *S*. *flavescens* extracts could induce CYP1A, CYP2B1/2, CYP2C11, and CYP3A in rats and mice [22]. The complexity of *S*. *flavescens* extracts may be the reason for varying experimental results, and while the alkaloids matrine and oxymatrine participated in inducting CYP isoforms [3]. Hence, clinicians should be aware of the in vivo concentration alterations of CYP1A2-metabolized substrates when their patients are administered *S*. *flavescens* with a high content of kurarinone.

Reaction phenotyping assays and chemical inhibition study evidenced that CYP1A2 and CYP2D6 play a significant role in catalyzing the formation of *P*_1_, whereas other enzymes exhibited limited ability to metabolize kurarinone. If kurarinone was coadministered with a CYP1A2 or CYP2D6 inhibitor, the CYP inhibition might likely amplify the blood concentration of kurarinone and lead to adverse effects.

Animals are often used as a model to predict kinetics and toxicity in humans. As we know, CYPs are classified into distinct subfamilies on the basis of amino acid sequence identity. A high degree of sequence identity does not necessarily indicate similar catalytic specificity, and a single amino acid substitution may even result in a change in substrate specificity. Therefore, the substantial differences in CYPs among various species may cause varying drug metabolism across species [34]. In the current experiments, enzyme kinetic studies were performed to further assess species difference. Differences in *K*_*m*_ value have shown significance to comprehend the basis of species-related differences in P450-catalyzed drug oxidation reactions [35]. Similar *K*_*m*_ values among various species reflect equivalent binding affinities toward the metabolic site and indicate species similarities [36]. The comparable *K*_*m*_ values for the formation of *P*_1_ in MLMs and HLMs in this study evidenced similar CYPs affinity in these two mammals. Monkeys and humans have a common ancestor since approximately 25 million years ago. Monkeys are genetically and physiologically similar to humans and are therefore the most extensively used nonhuman primate in basic and applied biomedical research [37]. Owing to higher *V*_max_ values, the CL_int_ values in MLMs, RLMs, and PLMs were found to be much higher than those in HLMs, while all the differences were statistically significant, evidencing that these liver preparations transformed kurarinone more efficiently than that in HLMs. The *K*_*m*_, *V*_max_, and CL_int_ values for the oxidative metabolism of kurarinone in CYP2D6 and CYP1A2 were similar. The *K*_*m*_ values were found to be much higher for kurarinone metabolism in liver microsomes than those in CYP2D6 and CYP1A2, which may be due to the nonspecific binding of kurarinone to microsomal material. The kinetic constant values of single CYP in cDNA expression systems and multiple CYPs in liver microsomal systems are incomparable [38].

Compounds can be classified into high-clearance (>70% liver blood flow), low-clearance (<30% liver blood flow), and intermediate-clearance drugs on the base of CL_*H*_ value projected from the in vitro data. Here, kurarinone was categorized as a high-clearance drug in humans and pigs. After intravenous administration of 10 mg/kg kurarinone in six rats, the *t*_1/2_ was calculated, 1.81 h [32], proving that kurarinone could be cleared quickly in vivo.

Molecular docking was performed in this study to investigate the molecular mechanism of interactions between kurarinone and CYPs. The results exhibited that kurarinone interacted with CYP1A2 (Asn312 and Asp320), CYP2C9 (Asp301), and CYP2D6 (Gln244 and Ser304) through formation of H-bonds. Asn312 and Asp320 are significant residues in the substrate and inhibitor recognition regions of CYP1A2 [39, 40]. Gln244 and Ser304 in CYP2D6 are active site residues involved in hydrogen bond formation with substrates [41].

More than 80% of orally administered drugs are absorbed into the bloodstream through passive diffusion in the small intestines. Thus, to detect the intestinal absorption and bioavailability, the passive diffusion of a drug must be comprehended. PAMPA is one of the most widely used assays to predict transcellular passive absorption in vitro models [42]. In the present study, permeability of kurarinone was evaluated at pH 4.0 and 7.4, which corresponded to conditions in the stomach, small intestine, and plasma. The permeability of kurarinone was found to be moderate, indicating that kurarinone was absorbed in the stomach and small intestine.

In this study, Kurarinone was thus evidenced to strongly inhibit UGT1A1, UGT1A6, CYP1A2, and CYP2C9, which could lead to DDIs. Kurarinone metabolism showed significant differences among species. CYP1A2 and CYP2D6 played important roles to metabolize kurarinone. The PAMPA permeability of kurarinone was moderate at both pH 4.0 and 7.4.

## Figures and Tables

**Table 1:** Kinetic parameters of *P*_1_ produced from kurarinone in pooled CYP1A2, CYP2D6, MLMs, HLMs, PLMs, and RLMs.

	CYP1A2^*a*^	CYP2D6^*a*^	MLMs^*b*^	HLMs^*b*^	PLMs^*b*^	RLMs^*b*^
Km	9.45 ± 2.11	13.76 ± 3.09	21.02 ± 3.42	21.96 ± 4.93	49.91 ± 7.91	18.08 ± 3.91
*V*_max_	2.45 ± 0.25	2.78 ± 0.36	4.83 ± 0.14	1.56 ± 0.24	8.46 ± 0.74	3.34 ± 0.19
CL_int_	0.26	0.21	0.23	0.07	0.17	0.18

^*a*^ *K*_*m*_, *V*_max_, and CL_int_ are expressed in units of *μ*M, *μ*mol·min^−1^·nmol^−1^ CYP, and mL·min^−1^·pmol^−1^ CYP, respectively. ^*b*^*K*_*m*_, *V*_max_, and CL_int_ are expressed in units of *μ*M, nmol·min^−1^·mg^−1^ protein, and mL·min^−1^·mg^−1^ protein, respectively.

**Table 2:** PAMPA effective permeability (Pe) values for kurarinone and permeability markers.

Compound	Pe × 10^−6^ (cm/s)	
	pH = 4	pH = 7
Naproxen (high permeability marker)	5.38 ± 1.36	5.76 ± 1.75
Furosemide (low permeability marker)	0.06 ± 0.01	0.07 ± 0.02
Kurarinone	3.87 ± 1.39	2.14 ± 0.82

## Data Availability

The data used to support the findings of this study are available from the corresponding author upon request.
